# The Role of Hypoxia-Inducible Factor-1α (HIF-1α) in the Progression of Ovarian Cancer: Perspectives on Female Infertility

**DOI:** 10.3390/cells14060437

**Published:** 2025-03-14

**Authors:** Md Ataur Rahman, Maroua Jalouli, Sujay Kumar Bhajan, Mohammed Al-Zharani, Abdel Halim Harrath

**Affiliations:** 1 Department of Oncology, Karmanos Cancer Institute, Wayne State University, Detroit, MI 48201, USA; ataur1981rahman@hotmail.com; 2 Department of Biology, College of Science, Imam Mohammad Ibn Saud Islamic University (IMSIU), Riyadh 11623, Saudi Arabia; mejalouli@imamu.edu.sa (M.J.); mmylzahrani@imamu.edu.sa (M.A.-Z.); 3 Department of Biotechnology and Genetic Engineering, Faculty of Life Sciences, Bangabandhu Sheikh Mujibur Rahman Science and Technology University, Gopalganj 8100, Bangladesh; sujaybge@gmail.com; 4 Zoology Department, College of Science, King Saud University, Riyadh 11451, Saudi Arabia

**Keywords:** Hypoxia-Inducible Factor-1α (HIF-1α), ovarian cancer, angiogenesis, female infertility, tumor microenvironment

## Abstract

Hypoxia-Inducible Factor-1α (HIF-1α) is crucial in the progression of ovarian cancer, especially in influencing its tumor microenvironment and promoting pathogenic pathways that worsen female infertility. In hypoxic settings, HIF-1α is stabilized and activates the transcription of genes associated with angiogenesis, metabolic reprogramming, epithelial-to-mesenchymal transition, and therapeutic resistance. Angiogenesis and glycolytic reprogramming mediated by HIF-1 tumor proliferation, survival, and metastasis. Its dysfunction concurrently impairs ovarian homeostasis, undermining follicular growth, hormone synthesis, and the ovarian vascular network, consequently contributing to infertility. Moreover, HIF-1α induces persistent inflammation and oxidative stress, promoting an environment damaging to reproductive health. Due to its dual function in ovarian cancer growth and infertility, HIF-1α is a potential therapeutic target. Strategies including small molecule inhibitors and nanoparticle-mediated delivery of drugs possess the potential to reduce HIF-1α activity, hence reducing cancer progression while protecting fertility. This review seeks to clarify the molecular basis of HIF-1α in ovarian cancer and its effects on female infertility, providing insights into novel treatment approaches that target both controlling the disease and preserving fertility.

## 1. Introduction

**Figure 1:**
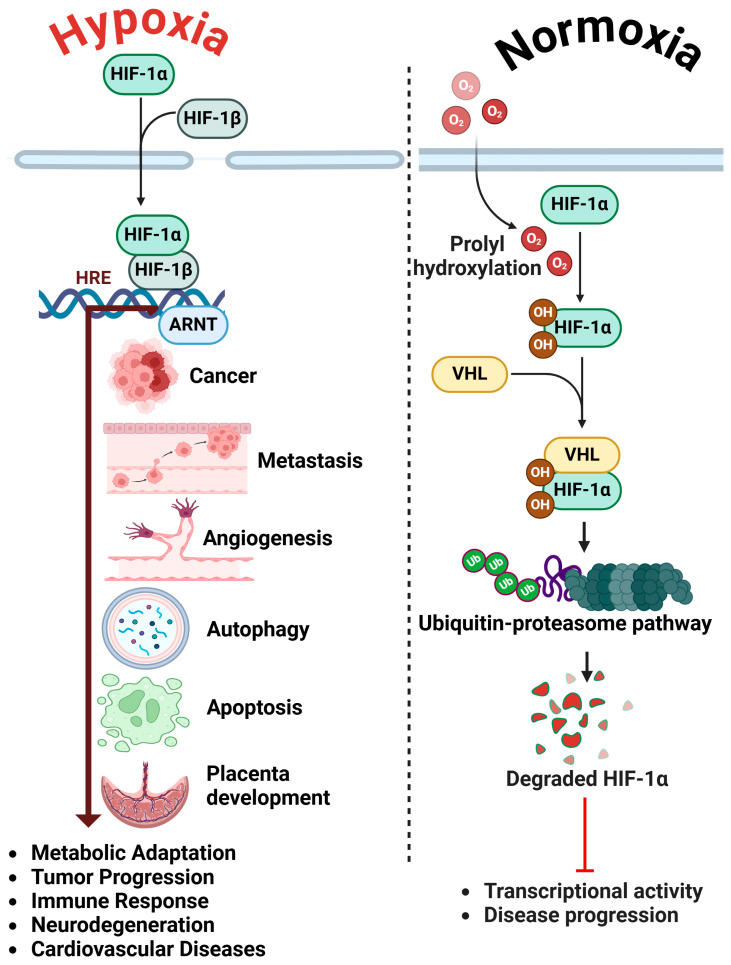
Hypoxia and Normoxia in HIF Signaling. During hypoxia (reduced oxygen levels), prolyl hydroxylase (PHD) activity is suppressed, leading to the stabilization of HIF-α. This stabilized HIF-α translocates to the nucleus and activates target genes associated with tumor progression, angiogenesis, metastasis, autophagy, apoptosis, metabolic adaptation, immune response, neurodegeneration, and cardiovascular diseases. Under normoxic conditions, HIF-α is hydroxylated by PHDs, resulting in its ubiquitination by the von Hippel-Lindau (VHL) complex and subsequent proteasomal degradation, hence inhibiting its transcriptional activity and influencing cellular adaptability and disease progression. The figure was created and modified using the BioRender online commercial platform.

HIF-1α functions as a transcription factor that modulates the expression of various genes essential for tumor growth, metastasis, and drug resistance [1]. In normoxic conditions, HIF-1α is subject to proteasomal degradation; however, in a hypoxic tumor microenvironment, it becomes stabilized and translocate to the nucleus, where it activates genes associated with angiogenesis, glycolysis, and epithelial-to-mesenchymal transition (EMT) [2]. These processes collectively allow ovarian cancer cells to survive in adverse conditions, avoid immune detection, and withstand conventional treatments [3]. In addition to its involvement in tumor progression, HIF-1α significantly impacts female reproductive health [4]. Ovarian function is significantly influenced by oxygen homeostasis. Disruptions in hypoxic signaling adversely affect follicular development, ovulation, and hormone production, which can lead to infertility [5]. In ovarian cancer, elevated HIF-1α levels can modify the ovarian vascular network, resulting in compromised blood supply to follicles and heightened oxidative stress, ultimately diminishing ovarian reserve and oocyte quality [6]. HIF-1α is essential for cellular adaptation to hypoxia and is crucial in the early stages of placental development. The regulation of angiogenesis, metabolism, and trophoblast function is essential for proper implantation and fetal nourishment [7]. Alterations in HIF-1α function are associated with pregnancy complications, including preeclampsia, intrauterine growth restriction (IUGR), and placental insufficiency, underscoring its critical role in fetal-maternal health (Figure 1) [8]. Additionally, HIF-1α-mediated inflammation intensifies damage to ovarian tissues, thereby reducing reproductive potential.

Ovarian cancer represents a significant threat among gynecological malignancies globally, frequently identified at advanced stages owing to its asymptomatic nature and the absence of effective early detection markers [9,10]. Ovarian cancer continues to exhibit high recurrence rates and poor prognosis, despite advancements in treatment modalities such as surgery and chemotherapy [11]. The tumor microenvironment, especially hypoxia, is a critical factor in the progression of ovarian cancer and its resistance to therapy [12]. Hypoxia, characterized by diminished oxygen levels, is a defining feature of solid tumors and significantly influences cancer progression through the induction of cellular adaptations that enhance survival, angiogenesis, metabolic reprogramming, and invasion (Figure 1) [13]. HIF-1α serves as a principal regulator of cellular responses to hypoxic conditions and has been thoroughly investigated for its role in cancer biology [14].

The relationship between ovarian cancer progression and infertility via HIF-1α signaling underscores the necessity for targeted therapeutic interventions. Ongoing research focuses on small-molecule inhibitors of HIF-1α, nanoparticle-mediated drug delivery systems for targeted inhibition, and metabolic reprogramming strategies to mitigate hypoxia-induced tumor aggressiveness [15]. Lifestyle and environmental factors that influence HIF-1α activity, including oxidative stress and dietary interventions, may provide supplementary strategies to enhance reproductive outcomes while addressing cancer progression [16]. Despite increasing evidence connecting HIF-1α to ovarian cancer and infertility, notable research gaps persist in elucidating its specific molecular mechanisms and potential therapeutic interventions. This review provides an analysis of HIF-1α’s role in ovarian cancer progression and its negative impact on female fertility. Elucidating these mechanisms may facilitate the development of novel therapeutic strategies that enhance cancer prognosis while preserving reproductive health in affected women.

## 2. HIF-1α in Ovarian Cancer Progression

**Figure 2:**
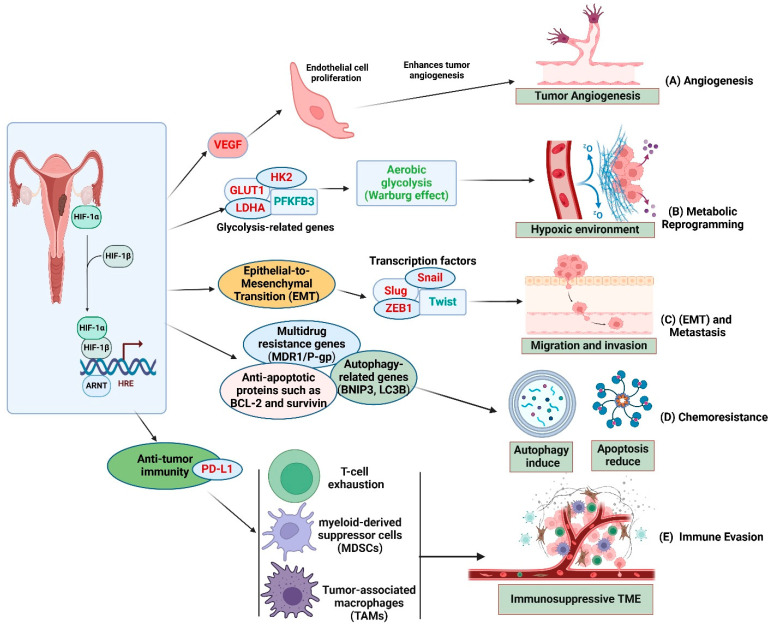
Molecular mechanism of HIF-1α signaling in ovarian cancer progression. HIF-1α migrates to the nucleus, where it dimerizes with HIF-1β and is associated with hypoxia-response elements (HREs) in the promoter regions of target genes. (**A**) Angiogenesis: HIF-1α upregulates VEGF, promoting endothelial cell proliferation and tumor angiogenesis, enhancing oxygen and nutrient supply. (**B**) Metabolic Reprogramming: HIF-1α induces glycolysis-related genes (GLUT1, HK2, PFKFB3, LDHA), shifting metabolism towards aerobic glycolysis (Warburg effect) for survival in hypoxia. (**C**) Epithelial-to-Mesenchymal Transition (EMT) and Metastasis: HIF-1α upregulates EMT regulators (Snail, Slug, Twist, ZEB1), downregulating E-cadherin and upregulating N-cadherin and vimentin, enhancing migration, invasion, and peritoneal dissemination. (**D**) Chemoresistance: HIF-1α promotes MDR1/P-gp, autophagy genes (BNIP3, LC3B), and anti-apoptotic proteins (BCL-2, survivin), reducing cisplatin-induced apoptosis. (**E**) Immune Evasion: HIF-1α induces PD-L1 expression, leading to T-cell exhaustion, and promotes MDSCs and TAMs, fostering an immunosuppressive tumor microenvironment (TME). The figure was created and modified using the BioRender online commercial platform.

The HIF-1α signaling pathway promotes ovarian cancer growth through the augmentation of angiogenesis, metabolic reprogramming, epithelial–mesenchymal transition (EMT), chemoresistance, and immune evasion (Figure 2) [17]. Targeting HIF-1α and its downstream pathways constitutes a viable approach for enhancing treatment outcomes in ovarian cancer. Thus playing a significant role in the aggressive characteristics of ovarian cancer [18].

### 2.1. Angiogenesis and Metabolic Reprogramming

Angiogenesis, the development of new blood vessels, is essential for tumor viability under hypoxic conditions. HIF-1α enhances angiogenesis mainly through the upregulation of vascular endothelial growth factors (VEGF), which is a significant angiogenic factor that encourages endothelial cell proliferation and the formation of blood vessels [19]. Elevated VEGF expression promotes the formation of an aberrant vascular network in ovarian tumors, thereby improving nutrient and oxygen supply to cancer cells [20]. Newly formed blood vessels frequently exhibit structural defects, resulting in irregular perfusion and ongoing tumor hypoxia, which in turn stabilizes HIF-1α and promotes tumor progression (Figure 2) [21]. Besides angiogenesis, HIF-1α is crucial for the metabolic reprogramming of ovarian cancer cells. In hypoxic environments, cancer cells transition from oxidative phosphorylation to aerobic glycolysis, a phenomenon referred to as the Warburg effect [22]. HIF-1α enhances the expression of glycolytic enzymes, including hexokinase (HK), lactate dehydrogenase A (LDHA), and glucose transporters (GLUT1), thereby promoting glucose uptake and lactate production [23]. This metabolic adaptation confers a survival advantage to ovarian cancer cells by sustaining ATP production and establishing an acidic microenvironment that facilitates immune evasion and enhances invasiveness (Figure 2).

### 2.2. Epithelial-to-Mesenchymal Transition (EMT)

Epithelial-to-Mesenchymal Transition (EMT) refers to the biological process whereby epithelial cells acquire mesenchymal characteristics, leading to increased motility and invasiveness [24]. This transition is crucial in various physiological and pathological contexts, including embryogenesis, wound healing, and cancer metastasis [25]. EMT is a vital mechanism in the metastasis of ovarian cancer, allowing epithelial cancer cells to adopt mesenchymal traits that facilitate increased motility and invasion. HIF-1α activates essential EMT transcription factors, including Snail, Slug, and Twist, leading to the suppression of epithelial markers, such as E-cadherin, and the induction of mesenchymal markers like N-cadherin and vimentin (Figure 2) [26]. This transition enhances the metastatic capability of ovarian cancer cells, facilitating their detachment from the primary tumor, invasion of adjacent tissues, and spread to remote organs. Additionally, HIF-1α-mediated EMT increases resistance to anoikis, a type of programmed cell death that takes place when epithelial cells detach from the extracellular matrix [27].

### 2.3. Resistance to Therapy

The development of resistance to chemotherapy and targeted therapies represents a significant challenge in the treatment of ovarian cancer. HIF-1α plays a role in therapy resistance via various mechanisms, notably by activating multidrug resistance (MDR) proteins, such as P-glycoprotein (P-gp) and breast cancer resistance protein (BCRP), which facilitate the efflux of chemotherapeutic agents from cancer cells [28]. HIF-1α also enhances DNA repair mechanisms and suppresses apoptosis through the upregulation of anti-apoptotic proteins, such as Bcl-2, thereby diminishing the effectiveness of conventional treatments [1]. These mechanisms underscore the essential function of HIF-1α in the progression of ovarian cancer. Targeting HIF-1α and its downstream pathways offers a promising therapeutic strategy to address tumor growth, metastasis, and drug resistance, thereby enhancing patient outcomes [29].

## 3. Impact of HIF-1α on Female Infertility

**Figure 3:**
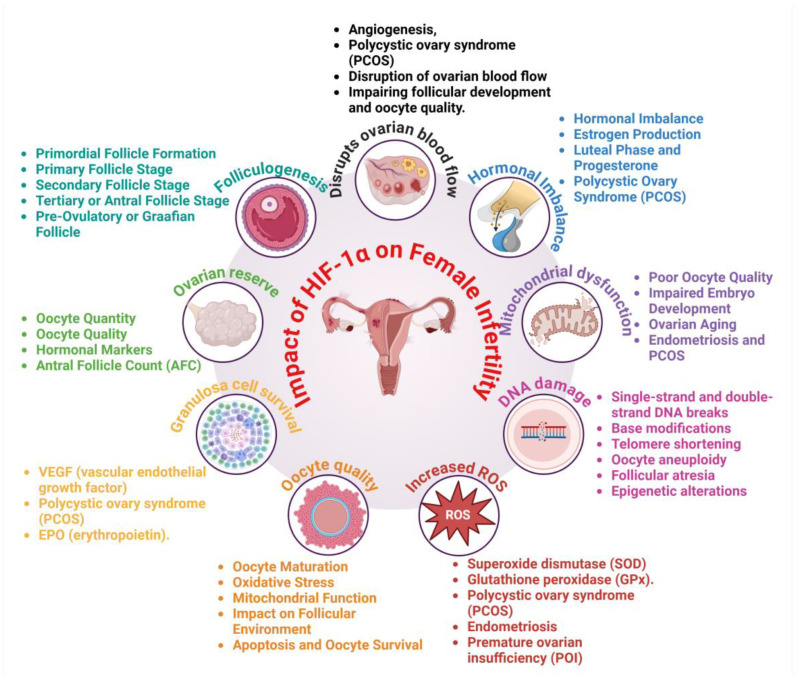
Impact of HIF-1α on Female Infertility. Dysregulated folliculogenesis, reduced ovarian reserve, granulosa cell apoptosis, and impaired oocyte quality. Elevated ROS, DNA damage, mitochondrial dysfunction, and increased pro-inflammatory cytokines (TNF-α, IL-6, IL-1β) accelerate ovarian aging and fibrosis. Suppressed estrogen synthesis (via aromatase inhibition), reduced AMH levels, impaired ovarian blood flow, and hypoxia-induced infertility. The figure was created and modified using the BioRender online commercial platform.

Ovarian cancer has a substantial effect on female reproductive health, with HIF-1α serving a critical function in the mechanisms associated with infertility [30]. HIF-1α serves as a key regulator of the hypoxic response, influencing tumor progression and impacting ovarian function through mechanisms such as angiogenesis, metabolic dysregulation, oxidative stress, inflammation, and hormonal imbalances [31]. These disruptions impair follicular development, oocyte quality, and overall reproductive capacity (Figure 3). Examining the role of HIF-1α in infertility is crucial for formulating targeted interventions to address reproductive dysfunction in women with ovarian cancer.

### 3.1. Disruption of Ovarian Function and Follicular Development

Ovarian function relies on a precise equilibrium of oxygen supply, hormonal signaling, and vascular integrity, all of which are governed by hypoxic responses [32]. Mild hypoxia contributes to follicular development under normal physiological conditions by stimulating angiogenesis and ensuring an adequate nutrient supply to growing follicles [33]. Excessive hypoxia resulting from HIF-1α overexpression in ovarian cancer disrupts the balance, impairing folliculogenesis and reducing ovarian reserve. HIF-1α modifies the ovarian microenvironment by disrupting the expression of essential growth factors for follicular maturation, such as vascular endothelial growth factor (VEGF), transforming growth factor-beta (TGF-β), and insulin-like growth factors (IGFs) [34,35]. Excessive VEGF production results in atypical blood vessel development in the ovaries, which disrupts follicular blood flow and induces localized hypoxia [36]. This leads to impaired folliculogenesis, reduced survival of granulosa cells, and heightened rates of follicular atresia. Excessive activation of HIF-1α also promotes granulosa cell apoptosis by increasing pro-apoptotic factors like Bcl-2-associated X protein (BAX) and decreasing anti-apoptotic protein expression, which accelerates follicular depletion [37]. Inadequate follicular development adversely impacts oocyte quality, resulting in a heightened risk of aneuploidy, diminished fertilization potential, and early embryonic arrest [38]. Moreover, changes in mitochondrial function caused by hypoxia lead to impaired ATP production, which elevates oxidative stress in oocytes and diminishes their developmental competence [39]. Consequently, women diagnosed with ovarian cancer frequently exhibit reduced ovarian reserve, decreased fertilization rates, and an increased risk of infertility [40].

### 3.2. Oxidative Stress and Chronic Inflammation

HIF-1α is crucial in the regulation of oxidative stress responses, with its overactivation in ovarian cancer resulting in an accumulation of excessive reactive oxygen species (ROS) [41]. ROS are produced during cellular metabolism and contribute to physiological signaling; however, their overproduction in hypoxic conditions can harm ovarian tissues, increasing the risk of infertility. Elevated levels of ROS disrupt folliculogenesis through the induction of DNA damage, lipid peroxidation, and mitochondrial dysfunction in ovarian cells [42]. Oxidative stress impacts granulosa and theca cells, resulting in premature ovarian aging and heightened follicular atresia [43]. Oxidative stress also disturbs the equilibrium of antioxidants like superoxide dismutase (SOD) and glutathione (GSH), increasing the susceptibility of ovarian cells to apoptosis [44].

Inflammation represents a significant factor modulated by HIF-1α in ovarian cancer. Chronic inflammation in the ovarian microenvironment leads to tissue fibrosis, impairing normal ovarian function [33]. HIF-1α increases the synthesis of pro-inflammatory cytokines, including tumor necrosis factor-alpha (TNF-α), interleukin-6 (IL-6), and interleukin-1 beta (IL-1β), thereby facilitating immune cell infiltration and oxidative stress [45]. The inflammatory environment hastens ovarian dysfunction, diminishes ovarian reserve, and adversely affects fertility. Chronic inflammation mediated by HIF-1α pathways is associated with endometriosis-like conditions in patients with ovarian cancer [46]. Endometriosis is defined by the growth of ectopic endometrial tissue, which is aggravated by hypoxic conditions, angiogenesis, and the production of cytokines [47]. An inflammatory and hypoxic ovarian microenvironment contributes to increased fibrosis and scarring, which further compromises ovarian function and results in reduced fertility rates.

### 3.3. Hormonal Imbalance and Vascular Abnormalities

Dysregulation of HIF-1α in ovarian cancer disrupts hormonal homeostasis, resulting in reproductive dysfunction. The hypothalamic–pituitary–ovarian (HPO) axis regulates reproductive hormones, such as follicle-stimulating hormone (FSH), luteinizing hormone (LH), estrogen, and progesterone [48]. HIF-1α disrupts this regulation by modifying hormone synthesis and receptor sensitivity in ovarian tissues [4]. The overexpression of HIF-1α significantly suppresses estrogen synthesis. HIF-1α suppresses aromatase, the enzyme that converts androgens to estrogen, resulting in reduced estrogen levels [49]. The hormonal imbalance adversely impacts endometrial receptivity and oocyte maturation, thereby diminishing the likelihood of successful implantation and pregnancy [50]. Furthermore, diminished estrogen levels are associated with ovarian insufficiency, thereby elevating the risk of premature ovarian failure in patients with ovarian cancer. HIF-1α influences the secretion of anti-Müllerian hormone (AMH), which serves as a marker of ovarian reserve [51]. Increased levels of HIF-1α are associated with diminished AMH expression, suggesting a decline in ovarian follicles and a shortened reproductive lifespan [52]. Vascular abnormalities caused by HIF-1α further contribute to infertility by impairing ovarian blood flow [53]. An irregular and dysfunctional vascular network hinders oxygen and nutrient delivery to follicles, resulting in hypoxic stress and heightened follicular atresia. Defective vasculature contributes to ovarian hyperstimulation syndrome (OHSS) in women undergoing assisted reproductive technologies (ART), complicating fertility treatments [54].

## 4. Clinical Implications and Potential Therapeutic Approaches

Ovarian cancer presents a considerable challenge owing to its late-stage diagnosis, aggressive progression, and elevated recurrence rates [55]. HIF-1α is crucial in the pathogenesis of ovarian cancer, facilitating angiogenesis, metabolic reprogramming, EMT, and resistance to therapy [4]. The dysregulation of this system has significant implications for female reproductive health, resulting in infertility due to oxidative stress, chronic inflammation, hormonal imbalances, and vascular abnormalities. Comprehending the clinical implications of HIF-1α establishes a basis for targeted therapeutic strategies that can enhance cancer prognosis while defending fertility. Various strategies, such as pharmacological inhibitors, antioxidant therapy, anti-inflammatory agents, nanoparticle-based drug delivery, and lifestyle modifications, have been identified as potential interventions to reduce HIF-1α-driven ovarian cancer progression and infertility.

### 4.1. Inhibitors of HIF-1α

Inhibiting HIF-1α with targeted agents offers a dual therapeutic strategy that effectively reduces ovarian cancer proliferation while safeguarding reproductive health. Numerous HIF-1α inhibitors have been investigated for their anti-cancer effects, with many demonstrating potential advantages in maintaining ovarian function (Table 1). Inhibition of HIF-1α represents a significant approach in cancer treatment, given its involvement in tumor growth, metastasis, and drug resistance. Multiple compounds that target HIF-1α via distinct mechanisms have been identified, providing therapeutic advantages in ovarian cancer.

PX-478 is a small molecule that inhibits HIF-1α transcription and protein accumulation. PX-478 inhibits angiogenesis and therapy resistance by reducing HIF-1α levels, thereby effectively suppressing the growth of ovarian cancer. Furthermore, PX-478 enhances ovarian vascular function, thereby preventing follicular atresia, which is a significant factor in female infertility [56]. Acriflavine inhibits the binding of HIF-1α to HIF-1β, thereby obstructing its transcriptional function. This disruption results in decreased cancer metastasis and angiogenesis, establishing it as an effective anti-tumor agent. Acriflavine also diminishes ovarian hypoxia, a significant factor contributing to follicular degeneration and suboptimal oocyte quality in women experiencing infertility [57]. Echinomycin disrupts HIF-1 DNA binding, inhibiting its ability to activate genes associated with tumor survival and metastasis. This leads to reduced aggressiveness of tumors in ovarian cancer. Echinomycin additionally safeguards ovarian follicles against oxidative stress, thereby promoting improved reproductive function [58]. Digoxin, commonly employed in the treatment of cardiac conditions, has been shown to reduce HIF-1α protein levels. It restricts ovarian tumor progression by inhibiting angiogenesis and cellular proliferation. Additionally, it enhances ovarian microcirculation, stabilizes hormonal balance, and mitigates reproductive dysfunction [59]. 2-Methoxyestradiol (2-ME) induces destabilization of the HIF-1α protein, thereby inhibiting its transcriptional influence on tumor growth. It has demonstrated potential in inhibiting cancer progression while concurrently restoring hormonal equilibrium and ovarian functionality in women experiencing infertility challenges [60]. YC-1 suppresses the synthesis of HIF-1α and the signaling of VEGF, both of which are critical in tumor vascularization and metastasis. The capacity to mitigate inflammatory damage in ovarian tissues positions it as a viable option for fertility preservation during ovarian cancer treatment [61]. LW6 facilitates the degradation of HIF-1α through the proteasome pathway, consequently restricting tumor adaptation to hypoxic conditions. Furthermore, it safeguards ovarian follicles from stress-induced apoptosis, thereby enhancing ovarian function and decreasing the likelihood of premature ovarian failure [62]. PX-12 inhibits the stabilization of HIF-1α under hypoxic conditions by targeting the thioredoxin system, thereby diminishing tumor resistance to chemotherapy. It restores oxidative balance in ovarian tissues, which is essential for preserving follicular integrity and reproductive potential [63]. KRIBB11 inhibits HIF-1α’s capacity to initiate gene transcription, thereby diminishing cancer cell invasion and resistance to therapy. It enhances ovarian follicular survival by mitigating hypoxia-induced stress, thus providing advantages for women facing reproductive issues [17]. Bortezomib facilitates the degradation of HIF-1α, thereby diminishing tumor adaptation to hypoxic conditions and improving the effectiveness of chemotherapy. Furthermore, it enhances ovarian cell viability, thereby decreasing the likelihood of infertility caused by chemotherapy [64].

**Table 1:** Inhibitors of HIF-1α present potential therapeutic strategies for targeting HIF-1α in the management of ovarian cancer and female infertility.

HIF-1α Inhibitor	Mechanism of Action	Potential Benefits for Ovarian Cancer	Potential Benefits for Female Infertility	Commercial Availability	Ref.
PX-478	Directly inhibits HIF-1α transcription and protein accumulation	Suppresses tumor angiogenesis, reduces therapy resistance	Improves ovarian vascular function, prevents follicular atresia	Yes	[56]
Acriflavine	Disrupts HIF-1α dimerization, blocking its transcriptional activity	Inhibits cancer progression and metastasis	Reduces hypoxia-induced ovarian dysfunction	Yes (Clinical use for infections)	[57]
Echinomycin	Binds to HIF-1 DNA binding sites, preventing target gene expression	Reduces hypoxia-driven tumor growth and survival	Protect ovarian follicles from oxidative damage	No (Investigational)	[58]
Digoxin	Downregulates HIF-1α protein levels via inhibition of its synthesis	Decreases cancer cell proliferation and vascular abnormalities	Improves ovarian microcirculation, enhances hormonal balance	Yes (FDA-approved cardiac drug)	[59]
2-Methoxyestradiol (2-ME)	Destabilizes HIF-1α protein and inhibits its transcription	Reduces angiogenesis and tumor progression	Restores hormonal equilibrium and ovarian function	No (Investigational)	[60]
YC-1	Inhibits HIF-1α synthesis and blocks downstream VEGF signaling	Suppresses tumor growth and hypoxia adaptation	Reduces inflammatory damage to ovarian tissues	No (Investigational)	[61]
LW6	Promotes HIF-1α degradation via the proteasome pathway	Decreases in hypoxia-induced drug resistance in ovarian cancer	Protect ovarian cells from stress-induced apoptosis	No (Investigational)	[62]
PX-12	Inhibits thioredoxin, preventing HIF-1α stabilization under hypoxia	Increases chemotherapy sensitivity, prevents metastasis	Restores oxidative balance in ovarian tissues	No (Investigational)	[63]
KRIBB11	Blocks HIF-1α transcriptional activation and nuclear localization	Reduces cancer cell invasion and therapy resistance	Improves ovarian follicular survival by limiting hypoxic stress	No (Investigational)	[17]
Bortezomib	Inhibits the proteasome, leading to degradation of HIF-1α	Prevents tumor adaptation to hypoxia, enhances drug efficacy	Enhances ovarian cell viability and prevents premature ovarian failure	Yes (FDA-approved for multiple myeloma)	[64]

The incorporation of HIF-1α inhibitors into clinical practice offers promising prospects for enhancing ovarian cancer outcomes while protecting fertility. Several critical areas necessitate additional investigation. Numerous HIF-1α inhibitors exhibit low bioavailability, necessitating nanoparticle-based delivery systems to improve stability and tissue penetration [65]. Additionally, the development of liposomal formulations, polymeric nanoparticles, and targeted drug carriers is essential for improved therapeutic outcomes [66]. Although numerous HIF-1α inhibitors have demonstrated potential in preclinical studies, additional clinical trials are required to evaluate their long-term impacts on ovarian function and fertility [4]. It is essential to ensure low toxicity, minimal off-target effects, and reproductive safety prior to widespread clinical application.

### 4.2. Antioxidant Therapy

Oxidative stress significantly results from HIF-1α overexpression in ovarian cancer and is essential in ovarian dysfunction. Accumulation of ROS leads to damage in ovarian follicles, diminishes oocyte quality, and accelerates premature ovarian aging [67]. Antioxidant therapy has emerged as a potential strategy to mitigate these effects, providing both anti-cancer and fertility-preserving advantages. Resveratrol is a polyphenolic compound present in grapes and berries, exhibiting significant antioxidant and anti-inflammatory properties. It directly inhibits the expression of HIF-1α and mitigates oxidative stress-induced DNA damage in ovarian tissues [68]. Preclinical studies indicate that resveratrol supplementation may enhance ovarian reserve and follicular health in cancer patients receiving chemotherapy. N-Acetylcysteine (NAC) serves as a precursor to glutathione, an essential cellular antioxidant that mitigates ROS and safeguards against oxidative damage. NAC has demonstrated the ability to suppress HIF-1α activity, inhibit tumor growth, and enhance chemosensitivity in ovarian cancer [69]. The capacity to restore redox balance in ovarian cells indicates a possible role in enhancing fertility outcomes. Melatonin functions as an effective free radical scavenger and has shown protective effects against oxidative stress in the ovaries. It regulates HIF-1α expression, diminishes VEGF-induced aberrant angiogenesis, and enhances mitochondrial function in oocytes [70]. Clinical studies indicate that melatonin supplementation improves oocyte quality and enhances reproductive success in women with ovarian dysfunction [71]. Antioxidant therapy shows potential in reducing the negative impacts of HIF-1α-induced oxidative stress. Further investigation is required to determine optimal dosing, timing, and combination strategies with existing therapies.

### 4.3. Anti-Inflammatory Agents

Inflammation significantly contributes to the progression of ovarian cancer by enhancing tumor survival, facilitating angiogenesis, and increasing resistance to therapy [72]. Inhibiting HIF-1α to target inflammation presents a promising strategy for enhancing treatment efficacy and decreasing tumor aggressiveness [73]. Chronic inflammation significantly contributes to the progression of ovarian cancer and infertility, with HIF-1α serving a pivotal role in the enhancement of pro-inflammatory cytokine production [45]. Targeting inflammatory pathways may reduce tumor aggressiveness and preserve ovarian function, playing a dual role in managing ovarian cancer progression through the inhibition of HIF-1α-driven inflammation and in the treatment of female infertility by safeguarding ovarian function and minimizing reproductive tissue damage (Table 2).

Curcumin, a polyphenolic compound derived from turmeric, inhibits the NF-κB and HIF-1α signaling pathways, leading to a reduction in inflammatory cytokines and oxidative stress. Curcumin inhibits tumor proliferation, diminishes angiogenesis, and improves chemotherapy efficacy in ovarian cancer. Curcumin mitigates inflammation, thereby preventing ovarian tissue damage and enhancing oocyte quality and reproductive outcomes [74]. Resveratrol, present in red grapes, inhibits HIF-1α and cytokine production, thereby decreasing tumor hypoxia and inflammation. It suppresses metastasis and chemotherapy resistance, positioning it as a promising candidate for ovarian cancer treatment. Resveratrol also maintains ovarian function by postponing ovarian aging and mitigating oxidative stress, thereby enhancing fertility potential [75]. Aspirin inhibits COX-2 and prostaglandin synthesis, thereby mitigating chronic inflammation linked to the progression of ovarian cancer. Research indicates that aspirin diminishes cancer cell proliferation and metastasis, establishing its role as an important chemopreventive agent. This also improves ovarian blood flow, mitigating infertility associated with endometriosis and promoting reproductive health [76]. Melatonin modulates the levels of HIF-1α, IL-6, and TNF-α, thereby mitigating inflammation induced by tumors. Melatonin inhibits tumor progression, enhances chemotherapy response, and decreases oxidative stress in ovarian cancer. Melatonin is essential for ovarian health, as it improves oocyte quality, safeguards ovarian follicles, and inhibits premature ovarian aging [77]. Sulforaphane, present in cruciferous vegetables, inhibits the activity of NF-κB and HIF-1α, leading to a reduction in the production of inflammatory cytokines. In ovarian cancer, it inhibits tumor growth, increases sensitivity to chemotherapy, and prevents metastasis. Sulforaphane enhances the stability of the ovarian microenvironment, mitigates oxidative stress, and improves fertility outcomes [78]. Omega-3 fatty acids influence pro-inflammatory cytokines, thereby decreasing oxidative stress in ovarian tissues. In ovarian cancer, tumor growth is suppressed, and immune response is enhanced. Omega-3 fatty acids improve ovarian response, promote follicular development, and mitigate inflammation-associated infertility [79]. Quercetin, a flavonoid derived from plants, inhibits HIF-1α, TNF-α, and NF-κB, thereby obstructing inflammation induced by hypoxia. In ovarian cancer, it diminishes tumor progression, inhibits epithelial–mesenchymal transition (EMT), and improves the effectiveness of chemotherapy. Quercetin protects ovarian follicles from inflammatory damage, thereby preserving ovarian function and fertility potential [80]. NAC enhances glutathione concentration, thereby diminishing oxidative stress and inflammation in ovarian tissues. In ovarian cancer, it improves the response to chemotherapy and inhibits tumor resistance. NAC prevents ovarian fibrosis, restores hormonal balance, and enhances overall reproductive health [81]. Boswellia Serrata inhibits 5-LOX and NF-κB, thereby reducing inflammation in ovarian cancer and reproductive tissues. It reduces tumor-associated inflammation, inhibits metastasis, and enhances the efficacy of chemotherapy. Boswellia enhances ovarian function and mitigates inflammatory damage in cases of infertility, thereby improving fertility outcomes [82]. Gingerol inhibits COX-2, TNF-α, and IL-6, thereby decreasing systemic inflammation. Gingerol inhibits tumor progression, enhances chemotherapy response, and reduces drug resistance in ovarian cancer. It also mitigates inflammation-related ovarian dysfunction, thereby enhancing reproductive health and fertility [83].

**Table 2:** Anti-inflammatory agents may serve as a potential therapeutic approach for targeting HIF-1α in the control of ovarian cancer and the management of female infertility.

Anti-Inflammatory Agent	Mechanism of Action	Potential Benefits for Ovarian Cancer	Potential Benefits for Female Infertility	Limitations	Ref.
Curcumin	Inhibits NF-κB and HIF-1α signaling, reducing inflammation and oxidative stress	Suppresses tumor growth, reduces angiogenesis	Protects ovarian follicles, improves oocyte quality	Poor Stability, Toxicity and Biocompatibility	[74]
Resveratrol	Downregulates HIF-1α and cytokine production, reducing hypoxia-driven inflammation	Inhibits metastasis, enhances chemotherapy sensitivity	Preserves ovarian function, delays ovarian aging	Stability and Aggregation, Blood–Brain Barrier (BBB) Penetration	[75]
Aspirin	Blocks COX-2 and prostaglandins, reducing chronic inflammation	Decreases cancer cell proliferation, lowers risk of metastasis	Improves ovarian blood flow, reduces endometriosis-related infertility	Rapid Clearance, Limited Targeting Efficiency, Poor Stability,	[76]
Melatonin	Acts as an antioxidant and anti-inflammatory agent, reducing IL-6 and TNF-α levels	Inhibits HIF-1α-mediated tumor progression	Enhances ovarian reserve, protects against oxidative stress	Limited Targeting Efficiency, Regulatory Challenges, Rapid Clearance and Short Half-Life	[77]
Sulforaphane	NF-κB and HIF-1α, reducing inflammatory cytokines	Prevents tumor progression and therapy resistance	Improves ovarian microenvironment, enhances fertility potential	Stability and Aggregation, Cost of Production, Toxicity and Biocompatibility	[78]
Omega-3 Fatty Acids	Reduces pro-inflammatory cytokines and oxidative stress	Suppresses tumor growth, improves immune response	Enhances ovarian response, reduces inflammation-induced infertility	Poor Targeting Efficiency, Short Circulation Half-Life	[79]
Quercetin	Inhibits HIF-1α and TNF-α, preventing hypoxia-driven inflammation	Reduces cancer progression and EMT	Protects ovarian follicles from inflammatory damage	Stability and Aggregation, Blood–Brain Barrier (BBB) Penetration, Limited Targeting Efficiency	[80]
N-Acetylcysteine (NAC)	Boosts glutathione levels, reduces oxidative stress and inflammation	Increases chemotherapy efficacy, protects normal cells	Prevents ovarian fibrosis, restores hormonal balance	Toxicity Concerns, Biodegradability and Clearance, Scalability and Cost	[81]
Boswellia Serrata (Frankincense)	Inhibits 5-LOX and NF-κB, reducing inflammatory responses	Lowers tumor-related inflammation, prevents metastasis	Supports ovarian function, reducing inflammatory stress on reproductive tissues	Manufacturing Challenges, Biocompatibility, Limited Targeting	[82]
Gingerol (from Ginger)	Suppresses COX-2, TNF-α, and IL-6, reducing systemic inflammation	Inhibits ovarian cancer progression, enhances chemotherapy response	Reduces inflammation-related ovarian dysfunction, improves reproductive outcomes	Limited Tissue Penetration, Possible Drug Resistance, Toxicity, Regulatory Challenge	[83]

Additionally, metformin, initially utilized as an anti-diabetic medication, has garnered interest in its anti-inflammatory and anti-cancer effects. It inhibits the stabilization of HIF-1α through the activation of AMP-activated protein kinase (AMPK), which results in reduced tumor growth and improved efficacy of chemotherapy [84]. Metformin enhances insulin sensitivity, diminishes oxidative stress, and safeguards ovarian function in patients with polycystic ovary syndrome (PCOS) and ovarian cancer [85]. Interleukin-6 (IL-6) inhibitors play a significant role as IL-6 is a crucial inflammatory cytokine that is upregulated by HIF-1α in ovarian tumors. Monoclonal antibodies targeting IL-6, including tocilizumab, demonstrate potential in mitigating tumor-associated inflammation and enhancing immune responses. IL-6 inhibitors may enhance fertility outcomes and preserve ovarian function by reducing inflammatory stress in the ovarian microenvironment. Non-Steroidal Anti-Inflammatory Drugs (NSAIDs), including aspirin and ibuprofen, have been investigated for their potential to lower ovarian cancer risk through the inhibition of cyclooxygenase-2 (COX-2), an important mediator of inflammation [86]. Research indicates that NSAIDs may enhance ovarian blood flow, decrease fibrosis, and improve follicular viability in women at risk for ovarian failure. Anti-inflammatory therapies demonstrate significant potential in addressing oncological and reproductive issues [87]. Long-term effects on ovarian physiology necessitate additional research to confirm safety and efficacy. Future research should prioritize the optimization of bioavailability, formulation, and clinical applications to achieve dual benefits in oncology and reproductive medicine.

### 4.4. Nanoparticle-Based Drug Delivery

Nanoparticle-based drug delivery systems present an effective approach for targeting HIF-1α, facilitating accurate and efficient drug administration while reducing systemic toxicity. Nanocarriers improve drug stability, bioavailability, and penetration into hypoxic tumor regions, presenting a promising strategy for ovarian cancer treatment. Furthermore, specific nanoparticles have demonstrated the potential to safeguard ovarian function, thereby contributing to both cancer management and fertility preservation [12]. Nanoparticle-based drugs enhance specificity and efficacy in targeting HIF-1α, offering a dual benefit in the management of ovarian cancer progression while protecting female fertility (Table 3).

Lipid-based nanoparticles that encapsulate HIF-1α siRNA effectively silence the gene associated with hypoxia adaptation in cancer cells. These nanoparticles inhibit tumor growth and enhance chemotherapy efficacy by decreasing HIF-1α expression. Lower HIF-1α activity mitigates ovarian hypoxia, thereby safeguarding follicular development and maintaining ovarian function from a fertility standpoint [88]. PX-478, a recognized inhibitor of HIF-1α, has been integrated into polymeric nanoparticles to enhance its delivery and bioavailability. These nanoparticles inhibit HIF-1α transcriptional activity, thereby reducing angiogenesis and tumor invasion. Furthermore, they enhance ovarian vascularization, thereby decreasing hypoxic stress that may adversely affect fertility [61]. Acriflavine serves as an effective inhibitor of HIF-1α dimerization, and its formulation in nanoparticles improves drug penetration within tumors. Disruption of HIF-1α function inhibits ovarian cancer metastasis. Additionally, it inhibits HIF-1α-induced follicular atresia, thereby promoting improved ovarian health and reproductive capacity [57]. Polymeric nanoparticles loaded with curcumin demonstrate anti-inflammatory properties and inhibit HIF-1α. Targeting HIF-1α and NF-κB signaling reduces inflammation in tumors and enhances sensitivity to chemotherapy. Curcumin nanoparticles also safeguard ovarian follicles against oxidative stress-related damage, thereby enhancing fertility outcomes [89]. Resveratrol, a natural antioxidant, has been encapsulated in lipid-based nanoparticles to enhance its bioavailability and therapeutic efficacy. It regulates HIF-1α signaling and mitochondrial function, thereby effectively inhibiting cancer progression. Resveratrol enhances oocyte quality, ovarian reserve, and reproductive outcomes, thus serving as a dual-benefit therapy [90]. Melatonin-loaded solid lipid nanoparticles demonstrate antioxidant properties and stabilize mitochondria, leading to a significant reduction in cancer aggressiveness induced by hypoxia. These nanoparticles protect ovarian tissue by decreasing reactive oxygen species (ROS) levels, minimizing apoptosis, and enhancing overall reproductive health [77]. NAC nanoparticles restore redox balance and inhibit HIF-1α signaling, thereby enhancing chemosensitivity in ovarian cancer cells. NAC nanoparticles improve ovarian function by mitigating oxidative stress, a significant contributor to infertility [91]. Liposomal formulations of doxorubicin facilitate targeted drug delivery to hypoxic tumor regions, thereby minimizing off-target toxicity. These nanoparticles effectively eliminate ovarian cancer cells while minimizing ovarian damage, thus reducing the risk of infertility post-chemotherapy [92]. Gold nanoparticles linked to HIF-1α inhibitors improve tumor-specific inhibition, reducing angiogenesis and tumor hypoxia. Furthermore, these nanoparticles enhance ovarian blood circulation, thereby preventing premature ovarian aging and maintaining fertility [17]. Selenium-based nanoparticles demonstrate antioxidant and anti-inflammatory effects, modulating HIF-1α pathways in ovarian cancer. They protect ovarian follicles against oxidative stress, improve ovarian function, and maintain hormonal equilibrium, thereby promoting reproductive health [93].

**Table 3:** Nanoparticle-based drugs for potential therapeutic approaches targeting HIF-1α in ovarian cancer control and female infertility.

No.	Nanoparticle-Based Drug	Type of Nanoparticle	Mechanism of Action	Potential Benefits for Ovarian Cancer	Potential Benefits for Female Infertility	References
1	HIF-1α siRNA Nanoparticles	Lipid-based	Silences HIF-1α expression, reducing tumor hypoxia	Inhibits tumor progression, enhances therapy response	Protects ovarian function by reducing hypoxia-induced damage	[88]
2	PX-478-Loaded Nanoparticles	Polymeric	HIF-1α inhibitor, blocks transcriptional activity	Suppresses angiogenesis, EMT, and drug resistance	Improves ovarian vascularization, reduces oxidative stress	[61]
3	Acriflavine-Encapsulated Nanoparticles	Liposomal	Disrupts HIF-1α dimerization, blocking its function	Inhibits tumor growth and metastasis	Prevents HIF-1α-induced follicular atresia	[57]
4	Curcumin Nanoparticles	Polymeric	Anti-inflammatory, inhibits HIF-1α and NF-κB signaling	Reduces inflammation, enhances chemotherapy efficacy	Protect ovarian follicles from oxidative stress	[89]
5	Resveratrol-Loaded Nanoparticles	Lipid-based	Antioxidant, modulates HIF-1α and mitochondrial function	Inhibits cancer cell proliferation, reduces therapy resistance	Improves oocyte quality and ovarian reserve	[90]
6	Melatonin-Conjugated Nanoparticles	Solid lipid	Antioxidant, stabilizes mitochondrial function, downregulates HIF-1α	Reduces hypoxia-induced cancer aggressiveness	Protects ovarian tissue from ROS and apoptosis	[77]
7	N-Acetylcysteine (NAC) Nanoparticles	Polymeric	Restores redox balance, inhibits HIF-1α signaling	Increases chemosensitivity, prevents tumor relapse	Enhances ovarian function and fertility outcomes	[91]
8	Doxorubicin-Loaded Nanoparticles	Liposomal	Chemotherapy drug, enhances drug delivery to hypoxic tumors	Targets hypoxic cancer cells more effectively	Reduces off-target toxicity to ovarian tissue	[92]
9	HIF-1α Inhibitor-Conjugated Gold Nanoparticles	Gold-based	Enhances targeted inhibition of HIF-1α in tumors	Suppresses angiogenesis and tumor hypoxia	Prevents premature ovarian aging by improving blood flow	[17]
10	Selenium Nanoparticles	Metal-based	Antioxidant, regulates oxidative stress and HIF-1α pathways	Protects against cancer progression, boosts immune response	Enhances ovarian follicle survival and hormonal balance	[93]

Nanoparticle-based drug delivery offers an innovative and efficient method for targeting HIF-1α in the treatment of ovarian cancer, while also addressing issues related to female infertility. The integration of HIF-1α-targeted nanoparticles with antioxidant therapy and reproductive medicine has the potential to significantly alter treatment paradigms for ovarian cancer as nanomedicine advances. Personalized approaches and advanced drug delivery systems may enable effective control of ovarian cancer while preserving reproductive potential, thereby enhancing quality of life and long-term health outcomes for affected women.

## 5. Limitations and Future Directions

### 5.1. Research Gaps and Emerging Trends

Although there is an increasing amount of evidence connecting Hypoxia-Inducible Factor-1α (HIF-1α) to the progression of ovarian cancer and female infertility, significant gaps persist in our comprehension of this relationship. Preclinical studies have established the involvement of HIF-1α in tumor angiogenesis, metabolic reprogramming, EMT, and therapy resistance; however, the translation of these findings into clinical applications poses considerable challenges. A significant limitation is the absence of extensive clinical trials assessing HIF-1α inhibitors in patients with ovarian cancer. Small-molecule inhibitors, such as PX-478 and YC-1, demonstrate potential in preclinical models; however, their long-term efficacy, specificity, and safety in human patients are still uncertain [94,95]. The potential side effects of HIF-1α suppression on normal ovarian physiology and fertility require further investigation.

Recent trends indicate that precision medicine and genetic profiling may improve the efficacy of HIF-1α-targeted therapies. Identifying patient subgroups with unique HIF-1α expression patterns may enhance treatment strategies and reduce adverse effects. Advancements in nanoparticle-based drug delivery present a promising approach to enhance drug bioavailability, minimize toxicity, and improve treatment specificity [96]. A significant research gap pertains to the dual influence of HIF-1α on ovarian cancer and fertility. Targeting HIF-1α may inhibit tumor progression; however, its involvement in normal ovarian function is intricate. Future research should aim to clarify the molecular mechanisms through which HIF-1α dysregulation impacts follicular development, oocyte quality, and ovarian reserve.

### 5.2. Integrating Cancer Treatment with Fertility Preservation Strategies

A major challenge in ovarian cancer management is the need to balance effective treatment with the preservation of fertility. Conventional cancer treatments, such as chemotherapy and radiation, frequently result in ovarian failure, thereby diminishing the likelihood of natural conception. Assisted reproductive technologies (ART), including ovarian tissue cryopreservation and in vitro maturation (IVM), present potential benefits for cancer survivors [97]; however, their success rates are inconsistent and often remain in the experimental phase across various contexts. Integrating HIF-1α-targeted therapies with fertility preservation strategies is essential to address this gap. Co-administration of antioxidants, such as resveratrol and melatonin, with cancer treatments may protect ovarian function and mitigate oxidative stress-induced damage [98]. Utilizing HIF-1α inhibitors alongside ovarian cryoprotection techniques may provide a more integrated strategy for enhancing reproductive outcomes in cancer patients [17]. Future research should investigate personalized treatment strategies that consider both oncological and reproductive outcomes. The integration of oncology, reproductive medicine, and molecular biology facilitates the development of innovative therapeutic strategies aimed at concurrently addressing ovarian cancer and preserving fertility, thereby enhancing the quality of life for affected women.

## 6. Conclusions

Hypoxia-Inducible Factor-1α (HIF-1α) is integral to the progression of ovarian cancer, facilitating angiogenesis, metabolic reprogramming, epithelial-to-mesenchymal transition, and resistance to therapy. In addition to its oncogenic effects, dysregulation of HIF-1α adversely affects female fertility by impairing ovarian function, increasing oxidative stress, triggering chronic inflammation, and disrupting hormonal balance. Examining the complex relationship between HIF-1α, cancer progression, and reproductive health is essential for formulating targeted therapeutic strategies that enhance cancer prognosis while safeguarding fertility. Various therapeutic strategies have been developed, such as HIF-1α inhibitors, antioxidant therapy, anti-inflammatory agents, and nanoparticle-based drug delivery systems [99]. Despite the significant potential of these strategies, clinical translation is hindered by a scarcity of human trials, possible side effects, and the intricate nature of ovarian physiology. Future research must prioritize the integration of cancer treatment with fertility preservation strategies, utilizing advancements in precision medicine, nanotechnology, and reproductive biomedicine. A multidisciplinary approach integrating oncology and reproductive medicine is crucial for enhancing treatment outcomes. Tailoring therapies to individual patient needs can effectively combat ovarian cancer while preserving reproductive potential, thereby enhancing both survival rates and quality of life for affected women. Ongoing research and collaboration in this field will facilitate the development of innovative, patient-centered therapeutic solutions.

## Data Availability

No new data were created or analyzed in this study.
